# Loss of LafB activity reverses daptomycin resistance in *E. faecium*

**DOI:** 10.1128/mbio.00715-25

**Published:** 2025-11-28

**Authors:** Suelen Scarpa de Mello, Bailey Schultz, Byoungsook Goh, Alexandra Grote, Terrance Shea, Jacob Muscato, Sungwhan F. Oh, Abigail L. Manson, Ashlee M. Earl, Suzanne Walker, Ilana L. B. C. Camargo, Michael S. Gilmore

**Affiliations:** 1 Massachusetts Eye and Ear Infirmary 1866, Boston, Massachusetts, USA; 2 Harvard Medical School, Microbiology Department, Harvard Medical School 1811 , Boston, Massachusetts, USA; 3 Center for Experimental Therapeutics and Reperfusion Injury, Department of Anesthesiology, Perioperative and Pain Medicine, Brigham and Women’s Hospital, Boston, Massachusetts, USA; 4 Department of Medicine, Northwestern University Feinberg School of Medicine 12244, Chicago, Illinois, USA; 5 Infectious Disease and Microbiome Program, Broad Institute of MIT and Harvard, Cambridge, Massachusetts, USA; 6 Biology Department, Bowdoin College 2050, Brunswick, Maine, USA; 7 Graduate Program in Immunology, Harvard Medical School 1811 , Boston, Massachusetts, USA; 8 University of São Paulo, Physics Institute 61755, São Paulo, Brazil; Universite de Geneve, Geneva, Switzerland

**Keywords:** daptomycin, *Enterococcus faecium*, glycolipids, lipoteichoic acid, antibiotic susceptibility

## Abstract

Infections caused by multidrug-resistant enterococci, particularly vancomycin-resistant *Enterococcus* (VRE), present significant therapeutic challenges. Daptomycin, a last-line treatment for VRE, often loses efficacy due to the emergence of resistance. In this study, we revealed the critical role of the *lafB* gene as a key determinant of daptomycin susceptibility and resistance in *E. faecium*. We showed that in the absence of a functional *lafB*, daptomycin-resistant mutants did not emerge *in vitro*, and derivatives of clinical daptomycin-resistant strains engineered to lack functional *lafB* were rendered even more sensitive to daptomycin than wild-type daptomycin-susceptible strains. These findings indicated that functional *lafB* is critical for key known mechanisms of daptomycin resistance, and mutations in *lafB* have phenotypic dominance to those that otherwise confer resistance. Therefore, inhibiting the activity of the *lafB* gene product is predicted to prevent or reverse resistance, offering a promising new strategy for extending the efficacy of daptomycin for treating enterococcal infections.

**IMPORTANCE:**

Daptomycin is one of the few remaining effective antibiotics for treating vancomycin-resistant enterococcal infections but is limited by the emergence of resistance during protracted therapy. Here, we show that without a functional *lafB* gene, daptomycin-resistant mutants do not arise under conditions where wild-type strains readily generate daptomycin-resistant mutants. Furthermore, we show that loss of function mutation of the *lafB* gene in daptomycin-resistant clinical isolates renders them more susceptible to daptomycin than wild-type *Enterococcus faecium*. This indicates that an effective small molecule inhibitor of LafB activity or *lafB* gene expression would be a useful adjunctive for extending and restoring the therapeutic utility of daptomycin.

## INTRODUCTION

Enterococci are important causes of multidrug-resistant hospital infection (1, 2), and rates of vancomycin-resistant *Enterococcus* (VRE) can be as high as 70% for *Enterococcus faecium* in some hospitals (3). Daptomycin (DAP) is a cyclic lipopeptide antibiotic that is currently understood to exert its antibacterial effects through two mechanisms: it inhibits cell wall synthesis by binding to lipid II, causing lipid clustering; and it induces membrane depolarization independently of cell wall components (4). Its activity is enhanced by interacting with phosphatidylglycerol (PG), prevalent in gram-positive bacteria. In the presence of calcium ions, DAP forms a complex with lipid II and PG, disrupting cell wall synthesis and leading to membrane depolarization. This action can trigger autolysis in susceptible bacteria, with any delayed membrane permeability being a secondary effect related to autolysis rather than direct pore formation (4). DAP is a critical last-line drug for VRE, but its efficacy is compromised by the emergence of resistance during therapy (5–8). Mutations in genes encoding the LiaFSR cell membrane stress response system or cardiolipin synthase involved in phospholipid metabolism are common routes to DAP resistance in *E. faecium* (9–14).

Lipoteichoic acid (LTA) is an important component of the gram-positive cell membrane. In *Enterococcus faecalis*, the enterococcal species in which LTA is most well studied, defects in LTA biosynthesis lead to reduced biofilm formation, virulence, and pathogenesis (15–17). Because of its unique structure, enterococcal LTA has also been considered as a potential vaccine candidate (18, 19).

In a previous study, we identified and characterized an unusual DAP-hypersusceptible *E. faecium* isolate from a suspected urinary tract infection (20). This new and unexpected vulnerability to DAP was traced to a mutation in the gene *lafB*, which encodes a glycosyltransferase involved in LTA biosynthesis (20). When exposed to DAP *in vitro*, this DAP-hypersusceptible isolate consistently reverted the initial missense mutation back to the wild-type (WT) sequence, restoring typical WT levels of DAP susceptibility before acquiring any additional mutations that confer resistance. This raised the possibility that the mutation in *lafB* may be phenotypically dominant to the effects of other mutations known to confer DAP resistance (20).

To determine whether the naturally occurring missense mutation in *lafB* (i) resulted in LafB loss of function, (ii) was phenotypically dominant to other known forms of DAP resistance, and (iii) could potentially lead to alternate mechanisms of DAP resistance that may be *lafB*-independent, we generated and examined the phenotypes of non-reverting deletion derivatives of *lafB* in both WT and DAP-resistant *E. faecium* backgrounds.

## RESULTS

### Loss of *lafB* function leads to DAP hypersusceptibility

To test whether the hypersusceptible phenotype we initially associated with a missense mutation in *lafB* (20) is caused by loss of LafB function, we deleted 116 codons out of a total 349 of the *lafB* reading frame by allelic replacement (21) to generate a non-revertible mutant of strain *E. faecium* SM1. Deletion of *lafB* recapitulated the DAP hypersusceptibility seen in the naturally occurring clinical isolate (20). Moreover, providing the *lafB* reading frame in *trans* restored the WT level of susceptibility, proving that the phenotype is ascribable solely to a loss of LafB function (Table 1).

**TABLE 1:** MICs of antibiotics tested in the *lafB*-mutant *E. faecium* strain, compared to hypersusceptible and WT strains

Strains	MIC (mg/mL)					
	DAP	Colistin	Polymyxin B	Bacitracin	Gentamicin	Vancomycin
*E. faecium* SM1	2	512	512	16	8	1
*E. faecium* SM1lafBt577c	**0.06^*a*^**	**128**	**64**	**2**	**4**	**0.5**
*E. faecium* SM1*ΔlafB*	**0.06**	**128**	**64**	**2**	**4**	**0.5**
SM1*ΔlafB* -pAT28::*lafB*	**2**	NT^*b*^	NT	NT	NT	NT

^*a*^ Bold values indicate a reduction in MIC observed in strains lacking wild-type lafB across all antibiotics tested.

^*b*^ NT, not tested.

We next examined whether the DAP-hypersusceptible *lafB* loss of function mutant possessed altered susceptibilities to other antimicrobials. To test this, we examined susceptibility to other antibiotics that target cell membrane components/cell envelope precursors (bacitracin and vancomycin), as well as cationic antibiotics including gentamicin, colistin, and polymyxin B, where changes to the highly polyanionic LTA polymers resulting from *lafB* loss of function may have an impact. In addition to conferring hypersusceptibility to DAP, mutation in *lafB* also rendered *E. faecium* about 10-fold more susceptible to polymyxin B and bacitracin, about fourfold more susceptible to colistin, and about twofold more susceptible to gentamicin and vancomycin (Table 1).

### Modification of *lafB* leads to the production of an aberrant LTA

**Fig 1:**
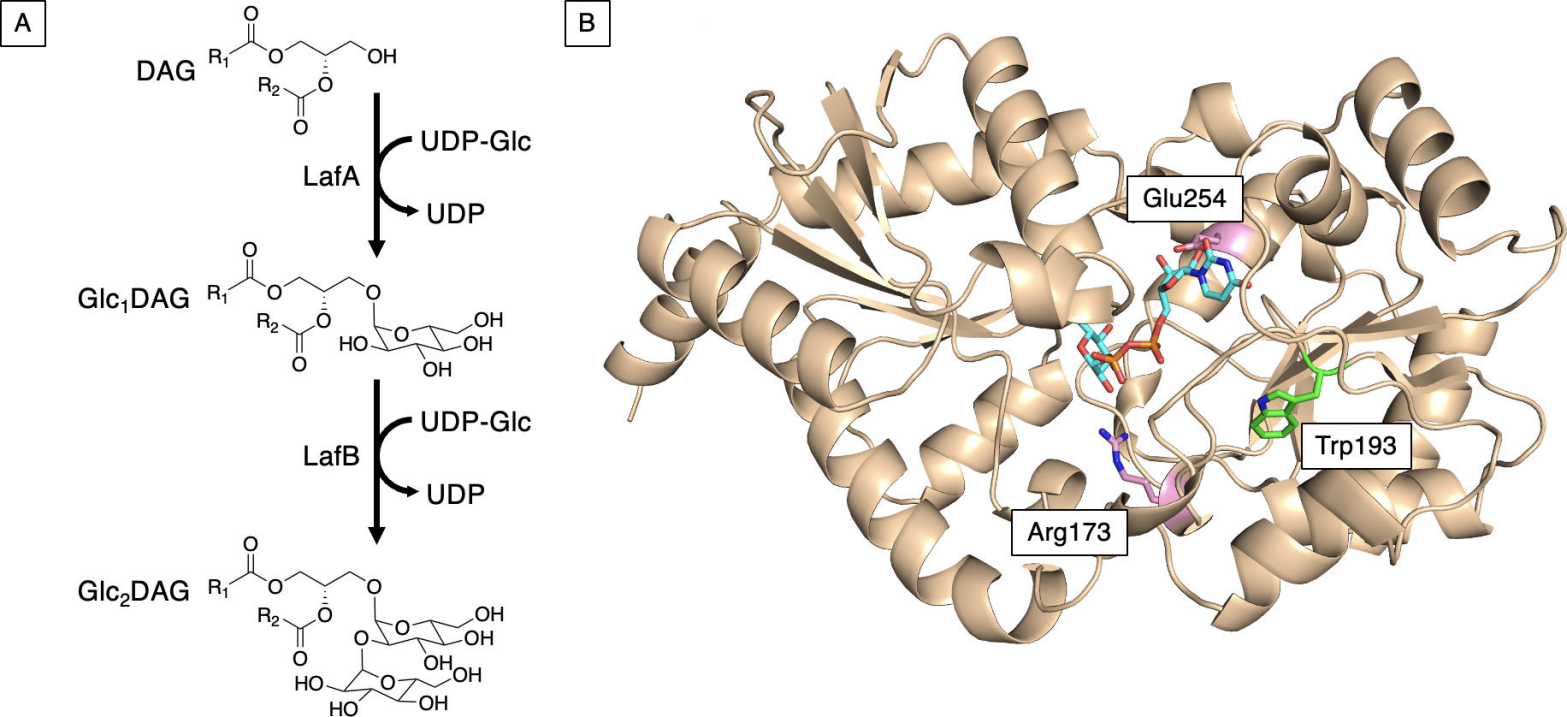
(**A**) Proposed pathway for LTA glycolipid biosynthesis in *E. faecium*. LafA transfers glucose onto diacylglycerol using UDP-glucose (UDP-Glc) as a substrate to make Glc_1_DAG. LafB then adds a second glucose to make an α-(1, 2)-linked Glc_2_DAG. (**B**) AlphaFold model of *E. faecium* LafB with UDP-glucose (cyan) modeled into the active site using the Chai Discovery-predicted LafB:UDP-Glc complex structure (23–25). Trp193 (green) is the WT residue replaced by an arginine as the result of a point mutation in the missense DAP-hypersusceptible strain. Chai and AlphaFold models of the protein were highly similar (RMSD: ~0.3 Å), and the predicted UDP-Glc binding site matched well with the UDP binding site for the *Thermosynechococcus elongatus* sucrose phosphate synthase crystal structure (PDBID: 6KIH). This sucrose phosphate synthase structure (26) was identified by the Dali server (27) as the experimental structure with the best structural match to the AlphaFold LafB structure (RMSD: ~2.4 Å). Glu254 and Arg173 (pink) are predicted to play roles in substrate binding based on homology to other similar glycosyltransferases.

To assess the functional impact of *lafB* mutation in *E. faecium*, we examined a DAP-hypersusceptible clinical isolate harboring a W193R substitution in the glycosyltransferase LafB. We previously showed that this mutation decreases solubility and thermal stability of the protein, consistent with a loss-of-function phenotype (22). This enzyme, homologous to BgsA in *E. faecalis*, catalyzes the second step in LTA anchor biosynthesis by converting monoglucosyldiacylglycerol (Glc_₁_DAG) to diglucosyldiacylglycerol (Glc_₂_DAG) (15) (Fig. 1A). Structural modeling using AlphaFold predicted that the W193R substitution lies within the nucleotide-binding domain, near the UDP-glucose binding pocket, and likely disrupts the hydrophobic core necessary for proper folding and stability (Fig. 1B).

**Fig 2:**
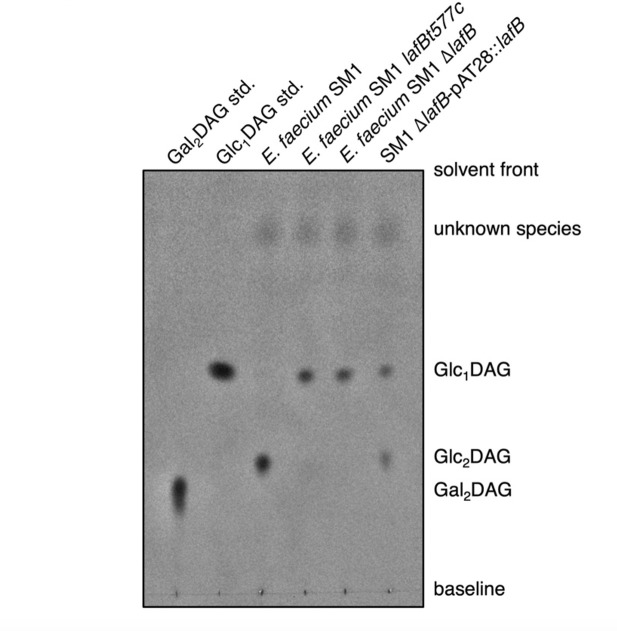
TLC analysis of glycolipids in total membrane lipid extracts from *E. faecium*. Glycolipid profiles of *E. faecium* SM1, SM1*lafB*T577C, SM1Δ*lafB*, and SM1 Δ*lafB*/pAT28::*lafB* mutants were compared to WT *E. faecium* SM1. Purified lipid standards included Gal₂DAG [1,2-diacyl-3-O-(α-D-galactosyl(1→6)-β-D-galactosyl)-sn-glycerol] and Glc_₁_DAG [1,2-diacyl-3-O-(α-D-glucopyranosyl)-sn-glycerol] (Avanti Polar Lipids). Glycolipid species Glc_₁_DAG, Glc_₂_DAG, and Gal_₂_DAG were identified based on migration relative to standards. Additional bands representing unknown lipid species are also indicated. The absence of glycolipid bands in the Δ*lafB* mutant and their restoration in the complemented strain demonstrates the role of *lafB* in glycolipid biosynthesis. Data are representative of three independent experiments.

**Fig 3:**
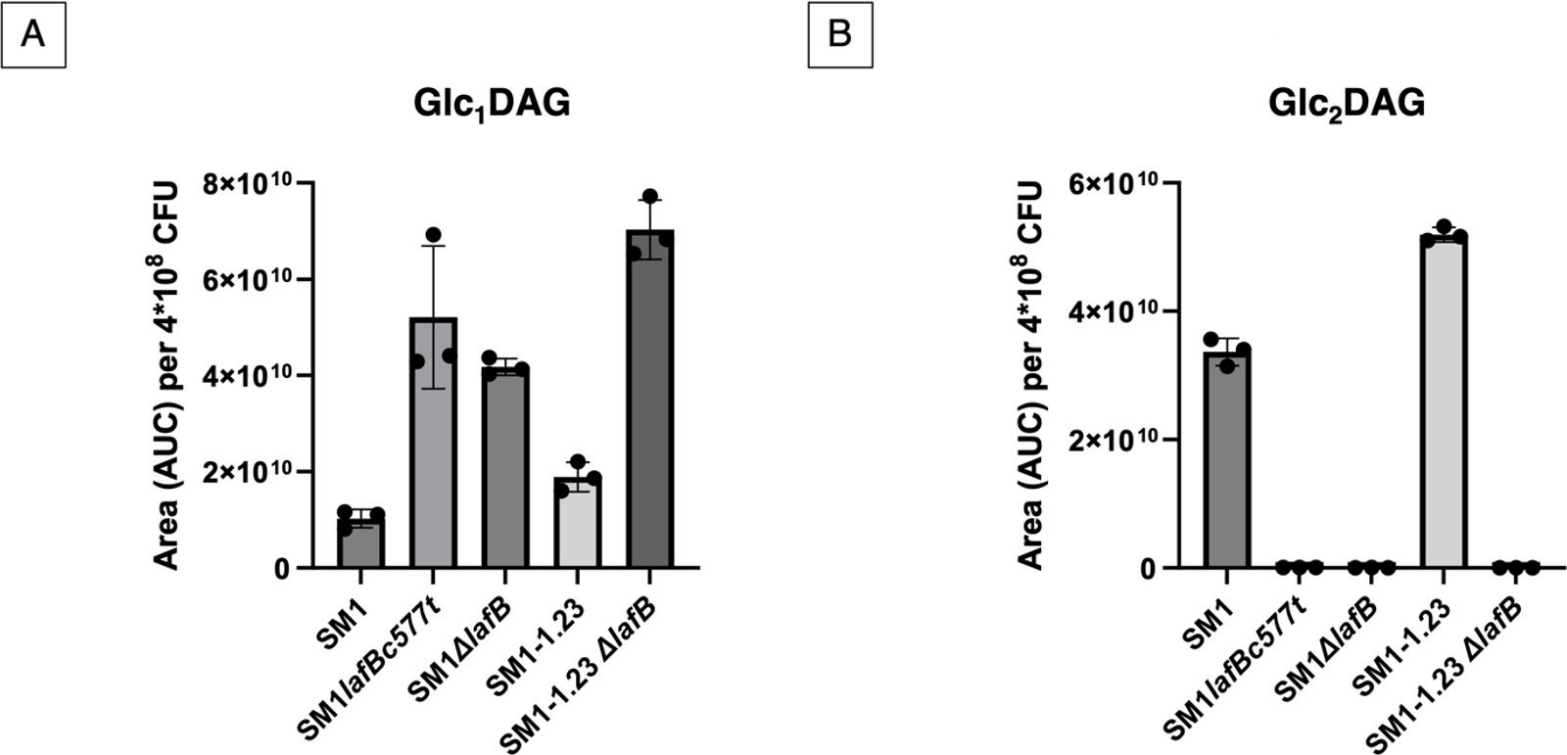
Quantitative comparison of glycolipids Glc1DAG (**A**) and Glc2DAG (**B**). The mean of the normalized area per 4 × 10^8^ CFU is shown. Peaks were quantified using extracted ion counts (EICs), and the mass (*m*/*z*) values used to generate these plots are provided in the supplementary material (Table S2 and S3). *E. faecium* SM1- wild-type strain; *E. faecium* SM1lafBt577c: clinical DAP hypersusceptible strain; *E. faecium* SM1ΔlafB - wild-type strain lacking lafB; *E. faecium* SM1-1.23(dak ^Ala432^Asp ^YycGAsp-272-Gly^ cls^His215Arg^): DAP-R evolved in vitro from wild-type strain; *E. faecium* SM1-1.23 (dak ^Ala432^Asp YycG^Asp-272-Gly^ cls^His215Ar^g) ΔlafB– evolved strain lacking lafB.

To determine the effect of *lafB* loss of function on *E. faecium* LTA biosynthesis, total lipids were extracted from the cell membranes of both WT *E. faecium* SM1 and *lafB* missense and deletion mutants and analyzed by thin-layer chromatography (TLC) with *N*-(1-naphthyl) ethylenediamine staining to detect glycolipids (Fig. 2). This revealed an accumulation of the monoglucosylated Glc_1_DAG produced by the product of *lafA* but no formation of the diglucosylated Glc_2_DAG product in both *lafB* mutants (SM1*lafBc577t* and SM1 *ΔlafB*). The accumulation of Glc_1_DAG, compared to SM1 and SM1-1.23, and the absence of Glc_2_DAG in the *lafB* mutants (SM1*lafBc577t*, SM1*ΔlafB*, and SM1-1.23*ΔlafB*) were confirmed by mass spectrometry-based lipidomic analysis (Fig. 3).

**Fig 4:**
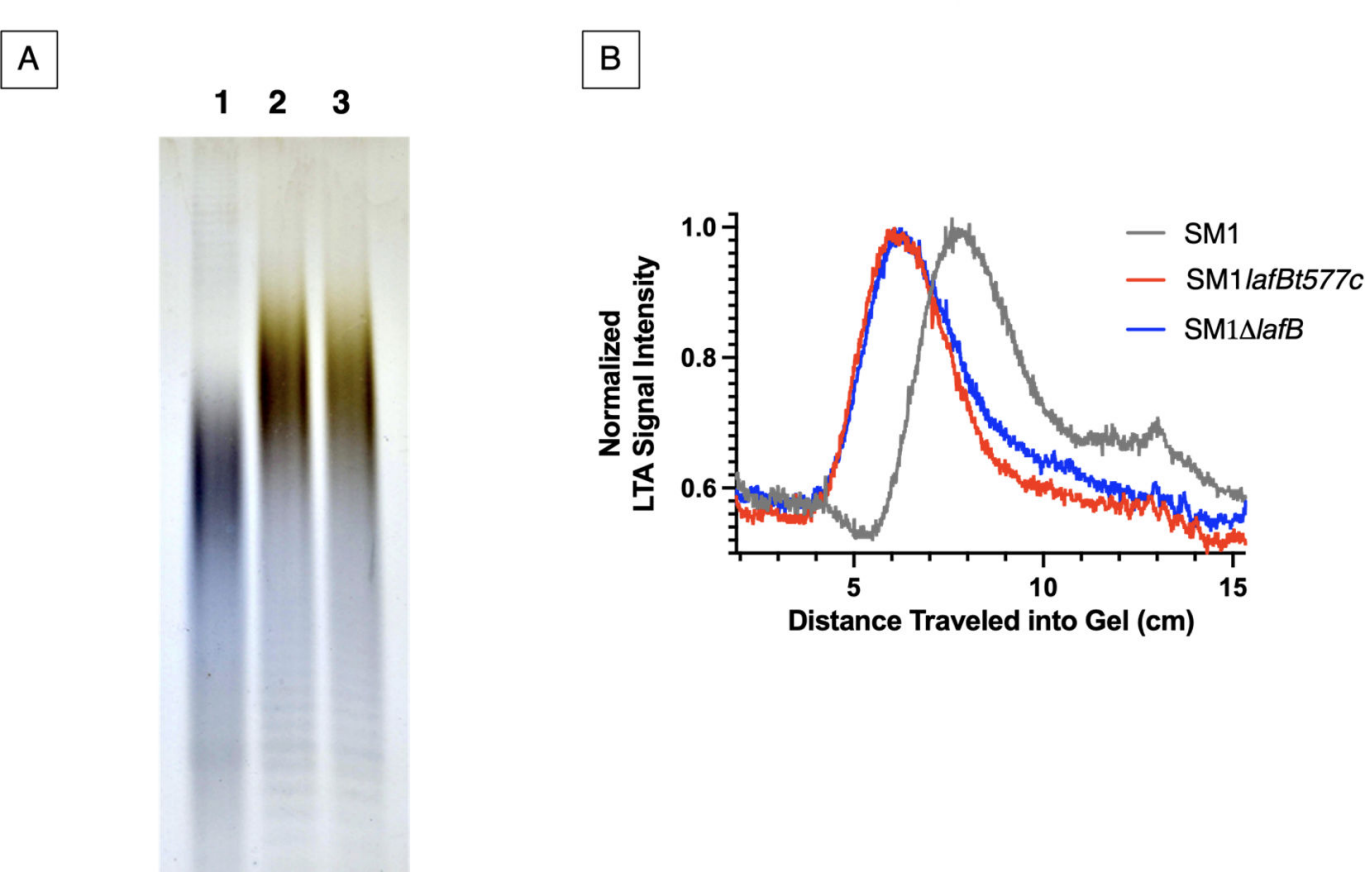
Altered migration of LTA in *lafB* mutant strains. (**A**) Alcian blue/silver-stained LTA isolated from WT *E. faecium* and *lafB* mutants, separated by electrophoresis through 20% polyacrylamide. Lane 1: LTA isolated from WT *E. faecium* SM1; Lane 2: LTA isolated from *E. faecium* SM1lafBt577c; Lane 3: LTA isolated from *E. faecium* SM1Δ*lafB*. The bulk of polymers extracted from *lafB* mutant lines migrate more slowly than those from the WT. Results shown here are representative of three independent experiments. (**B**) Densitometric analysis of Fig. 4A quantifies the leftward shift in polymer migration, reflecting altered size, structure, and/or composition compared to WT LTA.

Since an aberrant polymer of reduced mobility in polyacrylamide gel was observed previously in *E. faecalis* possessing a mutation in the homolog of *lafB* (15), we examined whether *lafB* inactivation in *E. faecium* led to the formation of a similar polymer. We compared the relative mobility of LTA purified from WT SM1 and the novel polymer produced by *lafB* mutants in polyacrylamide gels, following lipid tail removal by lipase treatment and visualization with Alcian blue/silver staining (Fig. 4A). As shown in Fig. 4B, loss of LafB functionality, whether due to a point mutation or deletion of a large portion of the reading frame, resulted in production of an aberrant polymer that migrated through polyacrylamide more slowly than the WT LTA polymer, similar to observations for *E. faecalis* (15). This indicates either a reduced charge-to-mass ratio or an increase in the size of the polymer produced when the typical Glc_2_DAG linker is unavailable.

### Defects in *lafB* block mutational pathways to DAP resistance

**Fig 5:**
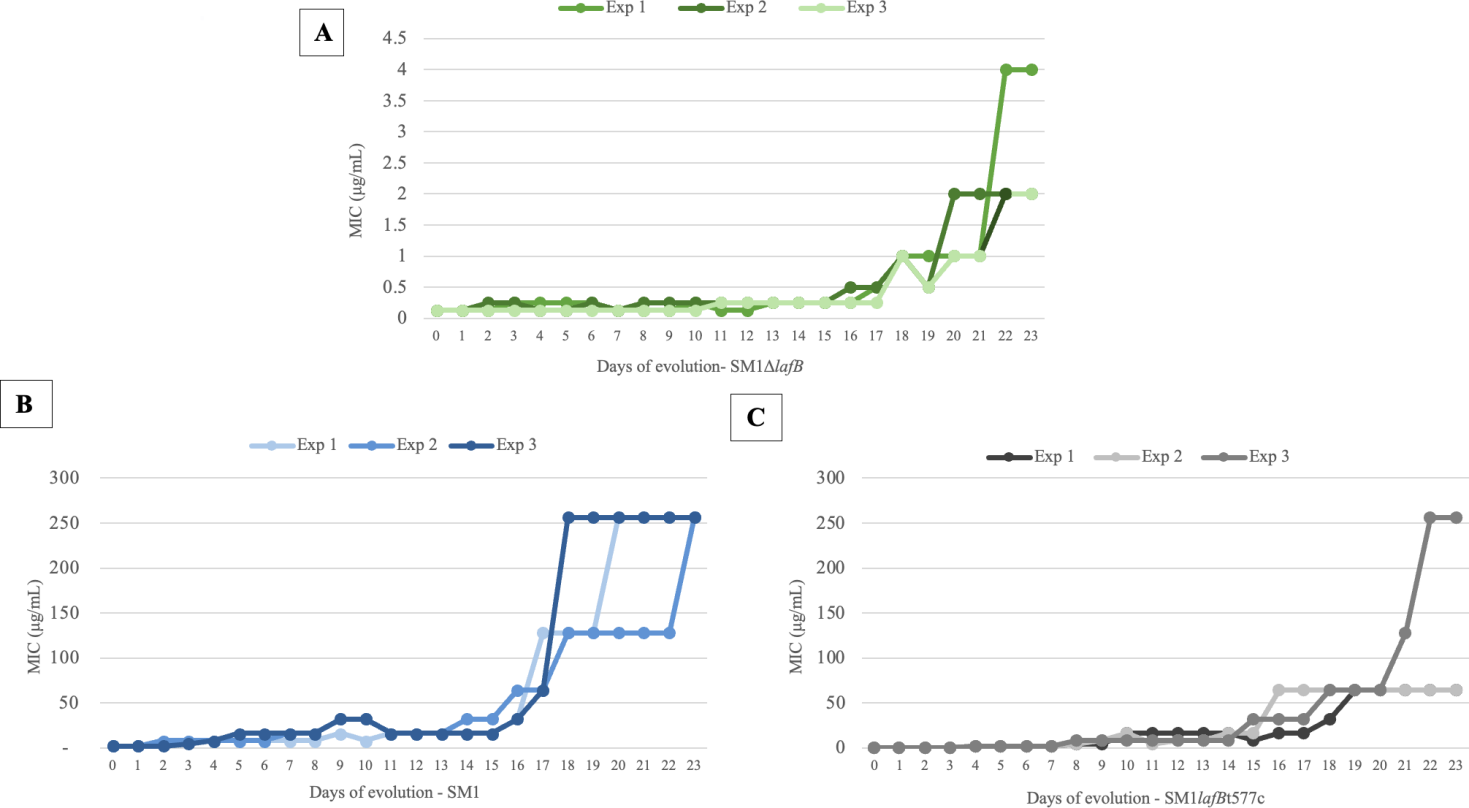
*In vitro* evolution of DAP resistance in *E. faecium* strains over 23 days. Graphs show changes in MIC values for *E. faecium* SM1Δ*lafB* (**A**), *E. faecium* SM1 (**B**), and *E. faecium* SM1*lafB*T577c (**C**) at each time point. Data represent individual wells in three biological replicates.

We next sought to determine whether pathways exist for the emergence of DAP resistance that are independent of a functional *lafB*. To do this, we exposed strains to stepwise increases in DAP concentration over the course of 23 days (Fig. S2; Table S1). As controls, the original clinical isolate possessing the missense point mutation in *lafB*, SM1*lafBt577c*, and its WT revertant SM1 were compared to the non-revertible *lafB* deletion strain SM1*ΔlafB*. Following extended stepwise exposure to DAP over 23 days, the *lafB*-deleted strain SM1*ΔlafB* never evolved resistance. In contrast, both control strains rapidly evolved mutants ultimately exhibiting over 100-fold increases in resistance to DAP (Fig. 5; Fig. S2; Table S1). Consistent with our prior work (20), the missense SM1*lafB*t577c loss of function control invariably reverted *lafB* to the WT sequence as the first step in the evolution of DAP resistance. This was followed by the appearance of a mutation in cardiolipin synthase (*cls*), a key enzyme in phospholipid biosynthesis (10, 11, 28–30) in one DAP-resistant derivative of SM1*lafB*t577c. In two out of the three replicates, DAP-resistant mutants derived from WT SM1 developed mutations in *cls* and in dihydroxyacetone kinase-like gene (*dak*), which encodes an enzyme that phosphorylates exogenous fatty acids for incorporation into membrane phospholipids (20, 31).

Three out of six replicates derived from both SM1 and SM1*lafB*t577c had mutations in YycG (EF1194, also known as WalK), which is predicted to function as a sensor histidine kinase within the YycF/YycG two-component system. YycG plays a crucial role in cell envelope biosynthesis in *Enterococcus* and is often mutated in DAP-resistant strains of *E. faecium* (13, 32–34). These three genes have all been implicated in enterococcal DAP resistance (Table 2). However, none of the mutations leading to high-level DAP resistance observed in WT or revertible *lafB* strains appeared in SM1*ΔlafB* over at least 115 generations.

**TABLE 2:** Genetic changes identified in DAP-resistant derivatives from SM1 and SM1*lafB*t577c

Parental strain	DAP	Annotation	Gene identification (*E. faecium* DO)	Amino acid change
	MIC range (mg/mL)			
SM1lafBt577c	32–256	cardiolipin synthase	HMPREF0351_11068	Ala-20-Asp
SM1	256			His-215-Arg
				Asn-13-Ser
SM1	128-256	DAK2 protein	HMPREF0351_10092	Ala-432-Asp
	256			Gly-94-Glu
SM1lafBt577c	64	YycG two-component system sensor histidine kinase	HMPREF0351_12361	Trp-609-STP
SM1	128–256			Asp-272-Gly
	64			Gly-564-Ser

To identify the genetic changes that could be associated with the small decreases in DAP susceptibility observed in SM1*ΔlafB*, we performed whole genome sequencing of those exhibiting the highest DAP minimum inhibitory concentration (MIC) levels. Derivatives of SM1*ΔlafB* were obtained exhibiting DAP MICs of 1–4 µg/mL, below levels of DAP resistance which is defined as an MIC greater than or equal to 8 µg/mL (35). Mutations in an alpha/beta hydrolase were identified in all three independently evolved SM1Δ*lafB* derived lineages (Table 3). A mutation in the LTA synthase (*ltaS*) gene, a transmembrane protein required to produce the polyglycerol-phosphate chain of LTA using PG as a substrate (36), occurred in two out of the three DAP mutant strains derived from SM1*ΔlafB* (Table 3). In *Staphylococcus aureus, ugtP* encodes the glycolipid synthase that catalyzes both glycosylation steps in Glc_2_DAG biosynthesis. In *ugtP* loss of function mutants, second site mutations in *ltaS* arise that generate shorter, less abundant LTA polymers presumably compensating for the effect of functional *ugtP* loss (37).

**TABLE 3:** Genetic changes identified in the DAP-evolved *E. faecium* SM1 strains

Isolate	DAP MIC (mg/mL)	Annotation	Gene identification (*E. faecium* DO)	Amino acid change
SM1ΔlafB 1-23 ^*a*^	4	alpha/beta hydrolase	HMPREF0351_11083	Gly-123-Glu
		HD protein	HMPREF0351_11978	Leu-673-Leu
		LTA synthase- ltaS	HMPREF0351_11744	Tyr-427-Ser
SM1ΔlafB 2-23^*a*^	2	alpha/beta hydrolase	HMPREF0351_11083	Asp-191-Tyr
		UDP-N-acetylglucosamine 1-carboxyvinyltransferase 2	HMPREF0351_12044	Cys-263-Arg
		LTA synthase- ltaS	HMPREF0351_11744	Tyr-427-Ser
SM1ΔlafB 3-23^*a*^	1	alpha/beta hydrolase	HMPREF0351_11083	Gly-32-Val
		phage tail tape measure protein	HMPREF0351_10868	Val-815-Glu

^*a*^ The first number represents the replicate and the second number the day of the experiment. (e.g., 1–23, replicate 1 day 23, last day of experiment).

### Deletion of *lafB* in DAP-resistant mutants restores susceptibility

To prove that *lafB* loss of function mutation was phenotypically dominant to mutations observed in clinical isolates that render them DAP-resistant, allelic replacement was used to delete *lafB* in an evolved DAP-resistant strain containing mutations in *dak*, *yycG*, and *cls* (strain SM1-1.23; MIC = 256 µg/mL), as well as in well-characterized DAP-resistant clinical isolates. In evolved DAP-resistant mutant SM1-1.23, deletion of *lafB* reduced the DAP MIC from 256 µg/mL to hypersusceptible levels of 0.125 µg/mL, comparable to the MIC of the *lafB* deletion made in the SM1 WT background. Thus, the additional mutations occurring in *dak*, *yycG,* and *cls* had no demonstrable effect in the absence of functional *lafB*. Moreover, DAP resistance could be partially restored by complementing the *lafB* mutant in *trans* with the WT *lafB* allele (Table 4).

**TABLE 4:** DAP MICs for *E. faecium* strains: *lafB* mutants, complemented strains, clinical isolates, and DAP-evolved mutants

Strains	Mutations	DAP MIC (mg/mL)
Clinical isolates		
HOU503	*liaR*^Trp73Cys^ *liaS*^Thr120Ala^	1–2
HOU503Δ*lafB*	Lacking *lafB*	0.06
R496	*liaF*^Ile142Thr^ *cls*^Asn13Ile^	16
R496Δ*lafB*	Lacking *lafB*	0.125
R496	Knockout restored via allelic replacement	16
R497	*liaS*^Thr120Ala^ *liaR*^Trp73Cys^ *cls ^insertion110^*^ofMetProLeu^	32
R497Δ*lafB*	Lacking *lafB*	0.125
R497	Knockout restored via allelic replacement	32
DAP evolved strains		
SM1-1.23	DAP-resistant mutant	256
SM1-1.23– Δ*lafB*	DAP-resistant mutant lacking *lafB*	0.125
SM1-1.23Δ*lafB*- pAT28	Empty plasmid	0.125
SM1-1.23-pAT28:*lafB*	Complementation of *lafB*	32
Controls		
SM1	WT	2
SM1lafBt577c	Clinical hypersusceptible strain	0.125
SM1*ΔlafB*	Lacking *lafB*	0.125
SM1*ΔlafB* -pAT28	Empty plasmid	0.125
SM1*ΔlafB* -pAT28::*lafB*	Complementation of *lafB*	2

Mutations in the *liaFSR* operon*,* especially the *liaR*^W73C^ variant, contribute to DAP resistance in clinical strains by constitutive induction of the cell wall stress response regulon (38). Given the importance of mutations in the *liaFSR* operon in clinical isolates, we investigated whether *lafB* is essential for this phenotype as well. Deletion of *lafB* decreased DAP MIC from 32 to 0.125 µg/mL in strain R496 (*liaF* ^I142T^ *cls* ^N13I)^, and from 16 to 0.125 µg/mL in strain R497 (*liaS*^T120A^ *liaR* ^W73C^ *cls ^insertion110^* ^of MPL^) (Table 4). For the clinical isolate HOU503, which is mutated in both *liaR* and *liaS* (LiaR^W73C^ and LiaS^T120A^), deletion of *lafB* decreased the DAP MIC from 1 to 0.06 µg/mL. These results show that depriving the cell of a functional LafB was phenotypically dominant to mutations in the *liaFSR* operon and to *yvcG*, *dak*, and *cls* genes, reverting DAP resistance driven by the most known resistance mechanisms (Table 4).

### Partial and complete complementation in *E. faecium* Δ*lafB* mutants

To validate that the observed phenotypes were specifically due to LafB loss and not other unknown mutations, we first complemented the SM1-23 Δ*lafB* null mutant with *lafB* expressed from the pAT28 plasmid as noted above. This construct partially restored DAP susceptibility, increasing the MIC from 0.125 to 32 µg/mL, but did not fully restore to the original SM1-23 MIC level of 256 µg/mL. The partial restoration is consistent with TLC results, which showed partial recovery of Glc_₂_DAG glycolipid production in the plasmid-complemented strain (Fig. 2), presumably stemming from either low-level expression despite the multicopy nature of the plasmid, or lack of potentially coordinate expression with other LTA biosynthetic functions. To achieve full complementation, we used allelic replacement to specifically restore only the *lafB* gene in strain R496 and R497 null mutant backgrounds. In both cases, this fully restored DAP resistance to MIC levels observed prior to *lafB* deletion. These results confirm that *lafB* is essential for Glc_₂_DAG biosynthesis and plays a critical role in modulating DAP susceptibility in both laboratory and clinical *E. faecium* backgrounds (Table 4).

### Exogenous oleic acid supplementation enhances DAP tolerance in WT but not *lafB* mutant

Previous reports found that *E. faecalis* can increase tolerance to DAP by incorporating exogenous fatty acids such as oleic acid which is abundant in human serum, into cell membrane lipids (39–41). To determine whether exogenous fatty acids could rescue the DAP hypersusceptible phenotype of *lafB* strains, we cultured WT and *lafB* mutant strains in the presence or absence of 20 mg/mL oleic acid. Supplementation of oleic acid to the growth medium (added exclusively in the MIC plate) resulted in a twofold increase in DAP tolerance for both WT and DAP-resistant strains, but no change in the DAP susceptibility was observed for *lafB* mutants (Table 5).

**TABLE 5:** Effect in DAP MIC when supplemented with 20 mg/mL of oleic acid^*b*^

MIC (mg/mL)		
Strains	DAP (mg/mL)	DAP + oleic acid (mg/mL)
SM1	2	4
SM1lafBt577c	0.125	0.125
SM1*ΔlafB*	0.125	0.125
SM1*ΔlafB* -pAT28::*lafB*	2	4
SM1*ΔlafB* -pAT28	0.125	0.125
SM1-1.23^*a*^	256	512
SM1-1.23- Δ*lafB*	0.125	0.125
SM1-1.23- Δ*lafB*- pAT28::*lafB*	32	64
SM1-1.23- Δ*lafB*- pAT28	0.125	0.125

^*a*^ *E. faecium* SM1-1.23 has the following mutations: *dak* ^Ala432Asp^ *YycG*^Asp272Gly^*cls*^His215Arg^.

^*b*^ Legend:* E. faecium* SM1*: *WT;* E. faecium* SM1*lafB*t577c: clinical DAP hypersusceptible strain; *E. faecium *SM1-1.23: DAP-R evolved *in vitro* from WT strain.

## DISCUSSION

Our previous work (20) raised the possibility that mutation of the *lafB* glucosyltransferase, and therefore loss of the α-(1→2)-linked second glucose on the diacylglycerol precursor of LTA, may be phenotypically dominant over key known mechanisms of DAP resistance in *E. faecium*. Here, we unambiguously prove this through several lines of work, showing that (i) non-revertible mutation of *lafB* prevents the emergence of DAP-resistant mutants under conditions where WT cells readily develop DAP resistance, (ii) deletion of *lafB* from either clinical or laboratory-derived DAP-resistant *E. faecium* strains carrying known resistance determinants reverses the resistance phenotype and renders the strains hypersusceptible, and (iii) in all cases tested, providing LafB functionality by *trans* complementation or allelic replacement partially or completely restores DAP resistance. In addition, the loss of LafB activity and associated alteration in LTA structure are accompanied by increased susceptibility to other cell wall-active and cationic antibiotics.

Loss of LafB activity led to a predicted absence of Glc_₂_DAG pools and accumulation of Glc_₁_DAG, consistent with disruption of the LTA glycolipid biosynthesis pathway, as previously observed in *E. faecalis* (15, 42). As in *E. faecalis*, mutants instead produced an altered form of LTA with reduced electrophoretic mobility. In *S. aureus* lacking a functional copy of *ugtP*, which catalyzes addition of both glucose units to DAG, analogously aberrant polymers are built on PG anchors and contain more glycerol-phosphate repeat units than WT LTA polymers (43). Whether the aberrant molecules observed in *E. faecium lafB* mutants or *E. faecalis bgsA* mutants (15) arise from a similar mechanism, potentially involving polymer assembly on the accumulating monoglycosylated DAG stem which is not available in *ugt* mutants of *S. aureus* remains unknown. Regardless, production of this altered polymer does not compensate for functional membrane changes that lead to DAP hypersusceptibility, even in strains that are otherwise highly DAP-resistant.

Loss of Glc_2_DAG production in *E. faecalis* has been linked to altered virulence in diseases such as endocarditis and peritonitis (17, 42, 44). By fundamentally changing the organization of the cell membrane, these disruptions likely cause pleiotropic effects, and the precise mechanisms by which defects in LTA biosynthesis contribute to loss of virulence remain unclear.

In sum, we demonstrate that *lafB* deletion prevents the emergence of DAP resistance, fully suppresses established DAP resistance phenotypes, and limits the potential effect of incorporation of exogenous fatty acids into membrane lipids on DAP susceptibility. These results highlight LafB as a promising therapeutic target for both blocking the emergence of DAP resistance during treatment and for restoring DAP susceptibility in resistant *E. faecium* infections. Additionally, this work demonstrates the pivotal role of LTA biosynthesis in modulating DAP susceptibility, offering new insights into the mechanisms underlying antibiotic resistance in this important pathogen.

## MATERIALS AND METHODS

### Genetic manipulation of *E. faecium*

A deletion mutant of *E. faecium* SM1 was created by allelic replacement of glycosyltransferase *lafB* gene. DNA segments upstream (741 bp) and downstream (738 bp) of the gene to be deleted were amplified by PCR with the following primers: F1 up-forward, R1 up-reverse; F2 down-forward and R2 down-reverse (Table S2). The PCR products from both regions were fused to generate a 1.479 bp PCR product lacking nucleotides 655–1004 of the *lafB* gene, which was then further digested using BamHI and PstI and ligated with BamHI/PstI-digested pLT06 (21) by using T4 DNA ligase (NEB). The ligation product was transformed into *Escherichia coli* EC1000 for propagation and sequence verification. The confirmed deletion construct was then introduced to *E. faecium* SM1 strain by electroporation (45). The SM1*ΔlafB* strain was obtained through temperature shifts and *p*-chlorophenylalanine counterselection, as previously described (21). Colonies were screened for *lafB* deletion by PCR using primers LafB_Int_F and LafB_Int_R (Table S2). The desired mutant was confirmed by Sanger sequencing. The strains used in this study are listed in Table S5.

To selectively repair the *lafB* deletion in the process of excluding other factors from contributing to the phenotypes observed, the full-length *lafB* gene along with its native promoter region was reintroduced into the *lafB* deleted strains of clinical isolates R496 and R497 by allelic replacement using the same methodology described above. The *lafB* gene was amplified by PCR using primers A and B (Table S2), generating a product that was cloned into the pLT06 vector digested with BamHI and PstI, followed by ligation and transformation into *E. coli* EC1000 for propagation and sequence verification. The verified construct was then electroporated into strains R496Δ*lafB* and R497Δ*lafB*. Single and double crossover recombinants were selected by the same temperature shifts and p-chlorophenylalanine counterselection procedure as above, and the presence of the restored *lafB* gene was confirmed by PCR and Sanger sequencing.

### Antimicrobial susceptibility testing

*E. faecium* strains were grown at 37°C in brain heart infusion (BHI) for all experimental conditions. Broth microdilution was employed to determine the MIC of DAP, polymyxin B, colistin, vancomycin, gentamycin, and bacitracin according to the Clinical and Laboratory Standards Institute guidelines, essentially as described (35). For assessing DAP susceptibility, cation-adjusted Mueller-Hinton broth was used and supplemented with 50 mg/L Ca^2+^ as recommended (35). In some assays, oleic acid (obtained from Sigma-Aldrich) was added to the broth to a final concentration of 20 µg/mL. All experiments were conducted in biological triplicates.

### Lipid extraction and analysis of glycolipid content by TLC

A modified Bligh-Dyer method (46, 47) was used to extract total lipids from the bacteria. Strains were streaked out from glycerol stocks onto BHI (from Hardy Diagnostics)-agar plates and grown at 30°C overnight. For each strain, 3 mL of BHI was inoculated with a single colony, and cultures were grown overnight at 30°C with aeration via shaking. Subcultures were then started with a 1:100 dilution of overnight culture into 50 mL fresh BHI media, and these sub-cultures were grown at 37°C, with aeration via shaking, until reaching an OD_600_ value of ~1.0–1.2. Sub-cultures were normalized to an OD_600_ value of 1.00, and then cells from 50 mL of the normalized sub-culture were pelleted by centrifugation at 10,000 × *g* for 10 min. Supernatant was removed, and the pellets were resuspended in 50 mL of 150 mM NaCl. Cells were then pelleted again by centrifugation at 10,000 × *g* for 10 min. Supernatant was discarded, and cell pellets were snap-frozen in liquid nitrogen and stored at −80°C for future use.

For lipid extraction, cell pellets were thawed on ice and then resuspended in 1 mL of 10 mM 2-(N-morpholino)ethanesulfonic acid (MES) (pH 6.5) with 150 mM NaCl. More of the same buffer was added to each sample to reach a total volume of 1.5 mL. This resuspension was then added to a glass tube containing 3.75 mL methanol and 1.875 mL chloroform. Samples were vortexed vigorously ~5 times each over the course of 10 min. Cell debris was pelleted by centrifugation at 2,000 × *g* for 10 min in a swinging bucket rotor. Supernatant was transferred to a new glass tube, to which 1.875 mL chloroform and 1.875 mL Milli-Q water was then added, giving a 1:1:0.9 final ratio of methanol:chloroform:water. Samples were again vortexed vigorously ~5 times each over the course of 10 min before centrifugation at 2,000 × *g* for 10 min in a swinging bucket rotor to separate the phases. The lower (organic) phase was carefully removed and transferred to a scintillation vial, and solvent was removed using a nitrogen dryer. Samples were then resuspended in 1 mL of chloroform and transferred to smaller glass vials for storage at −20°C.

For TLC analysis, solvent was fully removed from samples using a nitrogen dryer, and then samples were resuspended in 50 µL chloroform. Approximately 5 µL of each of the two standards (Gal_2_DAG: 1,2-diacyl-3-*O*-(α-d-galactosyl1-6)-β-d-galactosyl-*sn*-glycerol Avanti Polar Lipids 840524P, Glc_1_DAG: 1,2-diacyl-3-O-(α-D-glucopyranosyl)-sn-glycerol Avanti Polar Lipids 840522P) and ~15 µL of each sample was spotted on the baseline of a Silica gel 60 TLC plate. Once spots were fully dry, the plate was exposed to the running solvent (9:2 chloroform: methanol) in a pre-equilibrated beaker. When the solvent front neared the top of the plate, the plate was removed from the chamber and allowed to dry completely. The plate was then quickly dipped into the stain: 6.5 mM N-(1-naphthyl) ethylenediamine dihydrochloride (Sigma-Aldrich) in 97% methanol/3% concentrated sulfuric acid. Finally, the plate was placed onto a 100°C hot plate until spots appeared and then imaged using both a Nikon D3400 DSLR camera with an AF Micro-Nikkor 60 mm f/2.8D lens and an Azure Biosystems c150 imaging system. All experiments were conducted in biological triplicates.

### Isolation of LTA from *E. faecium*

This protocol was adapted from previous studies (37, 48–51). Strains were streaked out from glycerol stocks onto BHI agar plates and grown at 30°C overnight. For each strain, 10 mL of BHI media was inoculated with a single colony, and cultures were grown for 8 hours at 37°C with aeration via shaking. Large cultures were then started with a 1:200 dilution of the 8-hour culture into 1.0 L fresh BHI media, and these large cultures were grown at 30°C, with aeration via shaking, overnight (~16 hours). Cells were harvested by centrifugation at 5,000 × *g* for 20 min at 4°C. Supernatant was removed, and pellets were resuspended in phosphate-buffered saline (pH 7.4) to a total volume of ~48 mL. To the resuspension was added benzonase nuclease, MgCl_2_, lysozyme, and mutanolysin (Sigma-Aldrich M9901) to final concentrations of 10 U/mL, 5 mM, 1 mg/mL, and 10 U/mL in 50 mL total. Samples were tumbled end-over-end at 37°C for 1 hour, and then cells were lysed on an Avestin EmulsiFlex-C3 cell disruptor by passaging through ~8 times at 15,000 psi. Samples were then subjected to ultracentrifugation (140,000 × *g* for 45 min. at 4°C), and the supernatant was discarded. A Dounce tissue grinder (Wheaton) was then used to resuspend the membrane/cell debris material in 6 mL 50 mM sodium citrate (pH 4.7). More of the same buffer was then added to reach a final volume of 15 mL. At this point, tubes were snap-frozen in liquid nitrogen and stored at −80°C for future use.

For extraction of the LTAs, tubes were thawed, and then 15 mL of *n*-butanol was added. Samples were vortexed to mix and then tumbled at room temperature for 30 min, vortexing every ~5 min. Insoluble material was pelleted via centrifugation at 13,000 × *g* for 20 min at 4°C, and both layers of supernatant were moved together to a separate tube. Eight milliliters of the 50 mM sodium citrate (pH 4.7) buffer and 8 mL of *n*-butanol were added to the insoluble material for further extraction—vortexing followed by 30 min of tumbling at room temperature with more vortexing every ~5 min. These samples, plus the original supernatants in their separate tubes, were subjected to centrifugation at 13,000 × *g* for 20 min at 4°C. The upper organic layers were removed and discarded, and the lower aqueous layers were removed by pipetting through the insoluble interface and combined. An equal volume of *n*-butanol was added to the aqueous samples, and samples were vortexed to mix. Samples were then subjected to centrifugation at 13,000 × *g* for 15 min at 4°C. Upper organic layers were removed and discarded, and the lower aqueous layer was moved to a new tube. Once again, an equal volume of *n*-butanol was added to the aqueous layers, and the vortexing, centrifugation, and layer removal steps were repeated. 2.5 mL of methanol was added to the final aqueous layer samples (which now are ~12.5 mL), and samples were dried down by rotary evaporation to near-but-not-total dryness. Samples were stored in the glass flasks at −20°C overnight.

To isolate the LTAs, samples were dissolved in 5 mL of 5% isopropanol/95% water and then transferred to conical tubes. Insoluble material was pelleted by centrifugation at 3,200 × *g* for 10 min at 4°C. Samples were added to a pre-equilibrated (750 µL methanol, then 2 × 750 µL 20 mM sodium citrate, pH 4.7, with 5% isopropanol; all pushed through with air pressure) C18 Bakerbond SPE columns (silica packing material, 100 mg bed weight, 1 mL capacity, 40 µm particle size, 60 pore size) and allowed to flow through by gravity (note: this will take a few hours). For washing, air pressure was used to push liquid through columns that were washed twice with 900 µL of 20 mM sodium citrate, pH 4.7, with 5% isopropanol: then, sequentially, with 20 mM sodium citrate, pH 4.7, 15%/25%/35%/45%/65% isopropanol. Each wash was collected separately. All samples were dried down using a Vacufuge plus (Eppendorf) vacuum concentrator and stored at −20°C overnight.

The washes were resuspended in 100 µL of water each, tested for the presence of LTA by dot immunoblotting (next section), and then the 15%, 25%, 35%, and 45% isopropanol washes were combined based on the immunoblotting results. The combined washes were then snap-frozen in liquid nitrogen and then lyophilized for 2 days. Samples were resuspended in Milli-Q water to a final concentration of 50 mg dried sample per mL of water.

### Detection of LTA by dot immunoblotting

For each sample, 2.00 µL was spotted onto polyvinylidene difluoride (PVDF) membrane (pre-treated with methanol, then water, then Trans-Blot Turbo transfer buffer) that had been placed on top of a transfer buffer-soaked piece of extra thick blot/filter paper, which was itself on top of a dry piece of extra thick blot/filter paper and dry paper towels. The PVDF membrane was allowed to dry for ~5 min before sample spotting. After spotting, samples were allowed to soak into the membrane for 45 min, and then, the membrane was placed into a solution of 5% wt/vol milk powder in Tris-buffered saline with Tween 20 (TBST) to be rocked for 1 hour at room temperature. The membrane was then washed thrice with TBST for 5 min each time and then incubated in TBST with a 1:500 dilution of -LTA antibody (Hycult Biotech, mAb clone 55) for 1.5 hours at room temperature. The membrane was again washed thrice with TBST for 5 min each time and then incubated with a 1:1,000 dilution of mouse IgG, horseradish peroxidase (HRP)-linked antibody (Cell Signaling Technology 7076) for 1 hour at room temperature. Finally, the membrane was washed thrice with TBST for 5 min each before imaging by enhanced chemiluminescence with SuperSignal West Pico PLUS substrate (Thermo Scientific) on an Azure c600 gel/blot imaging system.

### Polyacrylamide gel electrophoresis analysis of *E. faecium* LTAs

This protocol was adapted from previous studies (37, 49, 50, 52, 53). Samples were prepared by adding 5.0 L 1.0 M Tris buffer (pH 7.5) to 20.0 L of 50 mg/mL LTA sample (note: the mass in the LTA sample is not purely from LTAs). The pH was then adjusted to ~8.0 via addition of 2.0 L 1.0 M NaOH and 4.0 L Milli-Q water. 1.0 L Resinase-HT (Strem Chemicals) was then added, and the samples were incubated overnight (~16 hours) at 50°C to allow for removal of the lipid tail acyl chains. Next, 1.0 L proteinase K (New England Biolabs) was added to remove protein contaminants, and samples were incubated for a further 5 hours at 50°C. Finally, samples were brought to room temperature, and 4.0 L of 100 mM AEBSF-HCl was added to quench the Proteinase K over a period of ~30 min.

A 20 cm × 20 cm (overall) polyacrylamide gel was cast with a 20% polyacrylamide resolving gel (with 6% of the total acrylamide added being bisacrylamide) and a ~2 cm 3% polyacrylamide stacking gel (with 3.3% of the total acrylamide being bisacrylamide) on top. Both portions of the gel were prepared with Tris pH 8.5 buffer (1.0 M final concentration for the resolving gel; 900 mM final concentration for the stacking gel), 0.1% wt/vol ammonium persulfate and 0.01% vol/vol tetramethylethylenediamine. The gel cassette was placed into the Protean II xi Cell electrophoresis system (Bio-Rad) along with 1.5 L total running buffer (100 mM Tris-tricine, pH 8.2). Samples were mixed with an appropriate amount of 3 loading buffer (50% vol/vol glycerol, 100 mM Tris-tricine pH 8.2, 0.02% wt/vol bromophenol blue), and 15 L per sample was loaded into the gel wells. The gel was run at a constant 35 mA current for ~20–24 hours.

To visualize the LTAs, the gel was first rinsed in Milli-Q water (three 5 min) and then stained in 1.0 mg/mL Alcian blue stain for at least 2 hours. Next, the gel was rinsed repeatedly (five 5 min) in Milli-Q water and then rinsed again for 2 hours and again overnight. Finally, the gel was stained according to the Silver Stain Plus kit instructions (Bio-Rad): 20 min incubation in 40% ethanol (vol/vol)/5% acetic acid (vol/vol) in water, two 10 min water incubations, and then incubation with the freshly mixed silver stain solution until LTAs are observable at the desired intensity. The silver stain was then removed, and the gel was treated with 5% acetic acid (vol/vol) in water for 15 min. Gel images were captured with a Nikon D3400 252 DSLR camera fitted with an AF Micro-Nikkor 60 mm f/2.8D lens. The line densitometry analysis was done in Fiji, and large spikes in intensity from visible and identifiable staining aberrations (dark spots/particles) were removed from the plots for clarity.

### DAP evolution assay

Single isolated colony of the *E. faecium* SM1 *ΔlafB* was used for each technical triplicate of an *in vitro* evolution experiment to generate increasingly resistant variants; the protocol was adapted from our previously published work (10). The evolution experiment started with overnight culture of each colony grown in BHI supplemented with 50 mg/L Ca^2+^ in 25 mL at 37 ˚C. After 22- to 24-hour incubation in 50 mL falcon tubes, cultures of each line were examined for visible bacterial growth. From these, 1 mL (10^9^ CFU/mL) of overnight culture was inoculated into tubes containing 25 mL of BHI supplemented with 50 mg/L Ca^2+^ and containing DAP at 1/2× MIC, 1× MIC, 2× MIC, and 4× MIC. All tubes were incubated at 37°C overnight with shaking (100 rpm). The following day, the falcon tube with visible growth at the highest DAP concentration was used as inoculum for the next series of tubes with increasing drug concentration. An aliquot of this growth was stored at −80°C for the remaining assays. This procedure was repeated for 23 days. The same strategy was applied for the hypersusceptible *E. faecium* SM1*lafB*t577c and *E. faecium* SM1 WT strains. The schematic representation is shown in Fig. S3.

### Whole genome sequencing and variant analysis

Total genomic DNA was extracted from cultures of each strain using the DNeasy Blood & Tissue Kit (Qiagen, Valencia, CA, USA).

Illumina sequencing libraries were prepared using the tagmentation-based and PCR-based Illumina DNA Prep kit and custom Integrated DNA Technologies (IDT) 10 bp unique dual indices with a target insert size of 320 bp. No additional DNA fragmentation or size selection steps were performed. Illumina sequencing was performed on an Illumina NovaSeq 6000 sequencer in multiplexed shared-flow-cell runs, producing 2 × 151 bp paired-end reads. Demultiplexing, quality control, and adapter trimming were performed with bcl-convert1 (v4.1.5).

The sample library for Oxford Nanopore sequencing of *E. faecium* SM1 was prepared using Oxford Nanopore Technologies Native Barcoding Kit 24 V14 (SQK-NBD114.24) to manufacturer’s specifications. The sample was run on a Nanopore R10.4.1 flow cell on a GridION. Samples were demultiplexed using Guppy (v6.3.8), with the “Super Accurate” base calling model (54).

A high-quality reference assembly for the genome of *E. faecium* HBSJRP18-2.7 was constructed using a combination of Illumina and Oxford Nanopore sequencing technologies. The hybrid assembly was performed using Unicycler v0.4.4 (55). The genome coverage was 317-fold (94-fold of Oxford Nanopore reads and 223-fold of Illumina reads).

Variants for the DAP evolved strains were called by first using BWA mem (version 0.7.12) (56) to align whole genome sequencing reads to the hybrid *E. faecium* SM1 reference genome and then using Pilon (version 1.12) (57) to call variants arising between timepoints. VCF files were filtered for variants containing the filter status PASS, and variants labeled as imprecise were removed. FastQ files were deposited under BioProject PRJNA1216963 NCBI’s SRA.

### LafB complementation

A construct in the shuttle vector pAT28 harboring a cloned *lafB* gene previously shown to be expressed, pAT28*lafB* (20), was transformed into *E. faecium* SM1*ΔlafB* to show that deletion of *lafB* in that recipient was solely responsible for its DAP hypersusceptible phenotype.

### Lipidomic analyses

Lipids were extracted from bacterial pellets using a mixture of methyl tert-butyl ether, methanol, and water (58). Perdeuterated (d35) beta-galactosylceramide (Matreya LLC) was added to each sample prior to extraction as an internal standard. Extracted lipids were analyzed by an ultra-high-performance liquid chromatography–tandem mass spectrometry (UHPLC-MS/MS) system, consisting of a Vanquish UHPLC and Q-Exactive Orbitrap (Thermo Scientific). Agilent Zorbax C18 column (4.6 mm × 75 mm × 1.8 µm, 600 µL min^−1^) was used for the lipid separation. The following LC gradient was used: 40% 2-propanol/40% acetonitrile/0.125% formic acid/10 mM ammonium formate isocratic for 2 min, then a linear change to 85% 2-propanol/10% acetonitrile/0.125% formic acid/10 mM ammonium formate over 3 min, and hold for 10 min, then return to the initial conditions over 0.1 min, and hold for 4.9 min. The column temperature was set at 40°C. The following heated electrospray ionization parameters were set: spray voltage, 3 kV; sheath gas, 60 AU; auxiliary gas, 15 AU; capillary temperature, 320°C; Aux gas heater temperature, 400°C; S-lens RF level, 65.0 AU. MS full scan was acquired from 300 to 1,600 *m*/*z* in positive ion mode (*R* = 70,000 at *m*/*z* 200). A top 3 data-dependent MS/MS was employed to obtain MS/MS spectra (*R* = 17,500 at *m*/*z* 200, normalized collision energy: 20 and 40 (AU), and isolation window: 1 *m*/*z*). Data processing, including peak alignment, lipid annotation, and peak quantitation, was performed with the MS-DIAL platform (59). The internal standard peak was quantified from each sample and used to calculate recovery values based on an external calibration curve. Peak areas of individual Glc_1_DAG and Glc_2_DAG were normalized by these recovery values to correct for variations in extraction and MS performance. Total areas for Glc_1_DAG and Glc_2_DAG were calculated by summing the normalized peak areas of the individual Glc_1_DAG and Glc_2_DAG species.

## SUPPLEMENTAL MATERIAL

Supplemental material mbio.00715-25-s0001.docx Supplemental figures and tables.

ASM does not own the copyrights to Supplemental Material that may be linked to, or accessed through, an article. The authors have granted ASM a non-exclusive, world-wide license to publish the Supplemental Material files. Please contact the corresponding author directly for reuse.
