# Supplementary material for: Loss of LafB activity reverses daptomycin resistance in E. faecium
Source: mBio. 2025 Nov 28;17(1):e00715-25. doi: 10.1128/mbio.00715-25 (PMC12802148; doi:10.1128/mbio.00715-25)
Supplement: Supplemental material — Supplemental figures and tables. [file mbio.00715-25-s0001.docx]

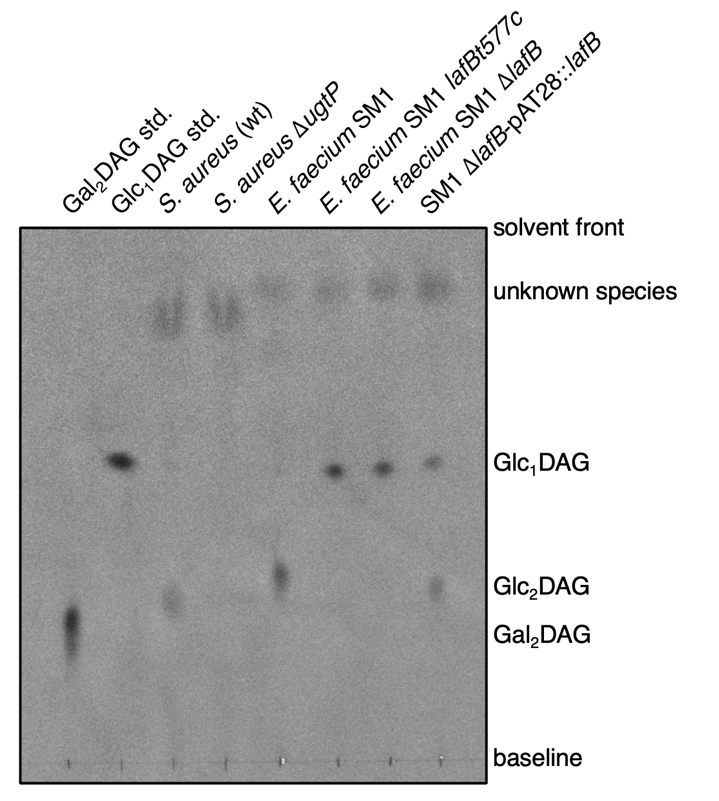


**Figure S1.** Glycolipid analysis of Enterococcus faecium membrane extracts by thin-layer chromatography (TLC). Total membrane lipids were extracted from E. faecium SM1, SM1*lafB*T577C, SM1Δ*lafB*, and SM1Δ*lafB*/pAT28::*lafB* and separated by TLC alongside purified standards Gal₂DAG [1,2-diacyl-3-O-(α-D-galactosyl-1→6)-β-D-galactosyl-sn-glycerol] and Glc₁DAG [1,2-diacyl-3-O-(α-D-glucopyranosyl)-sn-glycerol] (Avanti Polar Lipids). Additional controls include lipid extracts from wild-type Staphylococcus aureus and a Δ*ugtP* mutant lacking the glucosyltransferase responsible for Glc₂DAG synthesis. Glycolipid species Glc₁DAG, Glc₂DAG, and Gal₂DAG were identified based on migration relative to standards. Additional bands representing unknown lipid species are also indicated. The absence of glycolipid bands in the Δ*lafB* mutant and their restoration in the complemented strain demonstrate the role of lafB in glycolipid biosynthesis. As TLC-based glycolipid profiling has not previously been reported for E. faecium, the inclusion of S. aureus strains serves as a useful reference point for comparison to known glycolipid migration patterns. Data are representative of three independent experiments.

**
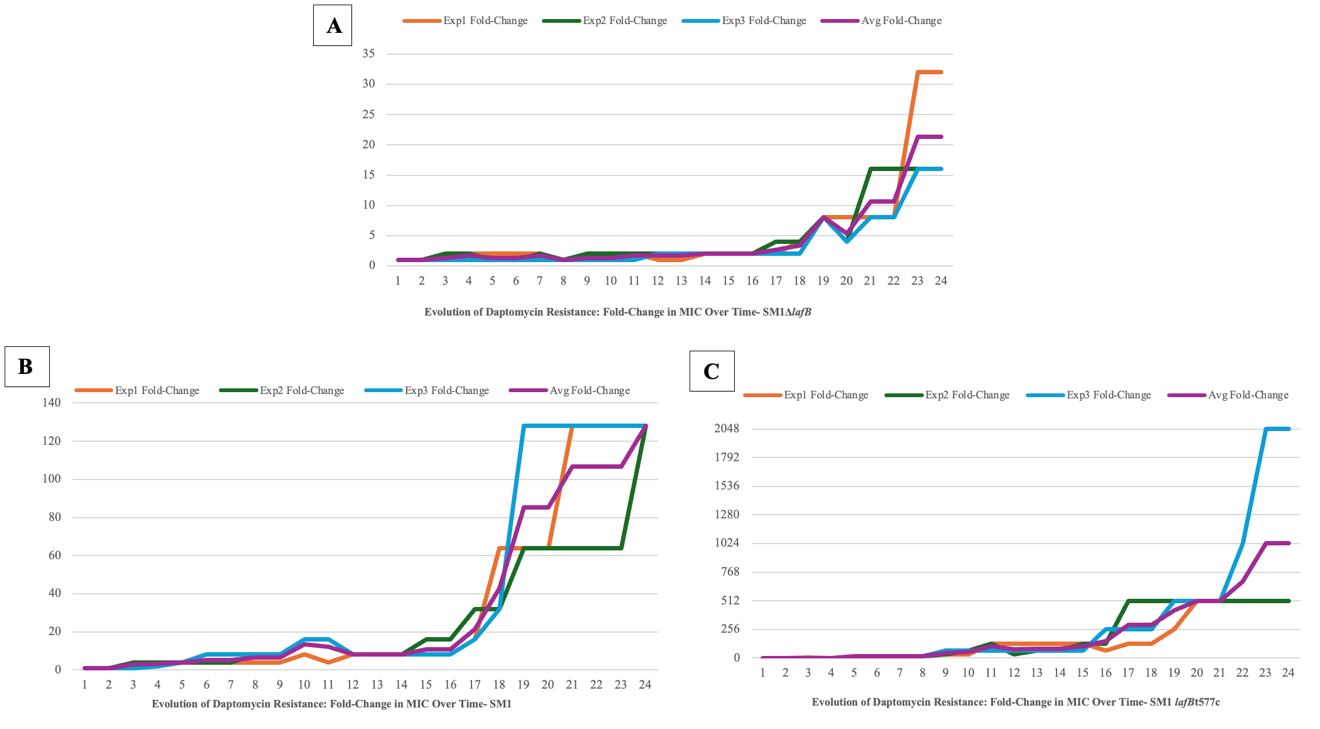
**

**Figure S2.** In vitro evolution of daptomycin resistance in E. faecium strains over 23 days. Graphs show fold-change in minimum inhibitory concentration (MIC) relative to Day 0 for E. faecium SM1ΔlafB (A), SM1 (B), and SM1lafBt577c (C). Each line represents an individual well from triplicate evolution experiments. Bold lines indicate the average fold-change in MIC across replicates at each time point.


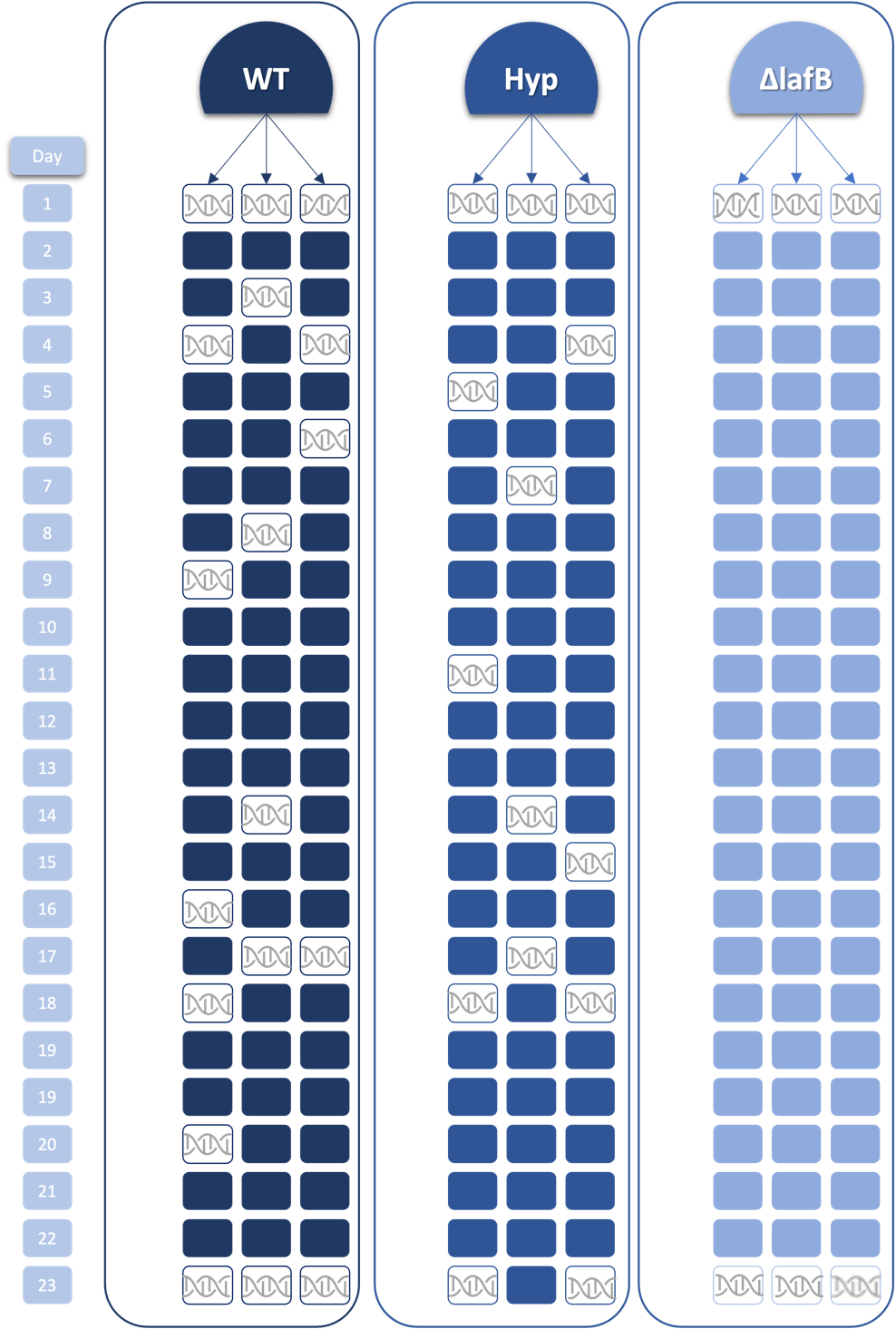


**Figure S3.** Schematic representation of the in vitro evolution of Enterococcus faecium under daptomycin selection. Strains SM1, SM1*lafB*t577c, and SM1ΔlafB were passaged in triplicate through increasing concentrations of daptomycin. Wells containing evolved populations selected for whole-genome sequencing are marked with DNA icons (DNA icon source, [Dimitry Miroliubov](https://www.flaticon.com/br/autores/dimitry-miroliubov)).

**Table S1.** Daptomycin MICs for evolved *E. faecium* derivatives sequenced. Strain names (SM1, SM1lafBt577c*,* and SM1*ΔlafB*) are followed by replicate number (first number) and the day of evolution (second number) corresponding to the time point at which each sequenced isolate was collected.

| **Strains** | **DAP MIC (mg/mL)** |
| --- | --- |
| SM1*lafB*t577c 1-0 | 0.125 |
| SM1*lafB*t577c 1-5 | 2 |
| SM1*lafB*t577c 1-11 | 16 |
| SM1*lafB*t577c 1-18 | 32 |
| SM1*lafB*t577c 1-23 | 128 |
| SM1*lafB*t577c 2-7 | 4 |
| SM1*lafB*t577c 2-14 | 16 |
| SM1*lafB*t577c 2-17 | 32 |
| SM1*lafB*t577c 3-4 | 2 |
| SM1*lafB*t577c 3-15 | 16 |
| SM1*lafB*t577c 3-18 | 64 |
| SM1*lafB*t577c 3-23 | 256 |
| SM1-1-0 | 2 |
| SM1-1-4 | 8 |
| SM1-1-9 | 16 |
| SM1-1-16 | 64 |
| SM1-1-18 | 128 |
| SM1-1-20 | 256 |
| SM1-1-23 | 256 |
| SM1-2-3 | 8 |
| SM1-2-8 | 16 |
| SM1-2-14 | 32 |
| SM1-2-17 | 64 |
| SM1-2-23 | 128 |
| SM1-3-4 | 8 |
| SM1-3-6 | 16 |
| SM1-3-17 | 128 |
| SM1-3-23 | 256 |
| SM1*ΔlafB* | 0.125 |
| SM1*ΔlafB* 1-23 | 4 |
| SM1*ΔlafB* 2-23 | 2 |
| SM1*ΔlafB* 3-23 | 2 |

**Table S2.** List of primers used in this study.

| **Oligo name** | **Sequence** | **Size (bp)** | **Reference** |
| --- | --- | --- | --- |
| **LafB_F1** | GGTGGTGGATCCGTCAGTAAGAATAGACA | 741 | This work |
| **LafB_R1** | CGGGTCGACGACATTTTCAGGATGATC |  |  |
| **LafB_F2** | CGGGTCGACAGTCTCTAGCAAATTA | 738 | This work |
| **LafB_R2** | GTTGTTCTGCAGCATTAATCAAGGCTTGG |  |  |
| **LafB_BamHI_F** | GGTGGTGGATCCGAGCCGTCCAATGGGTTC | 2327 | This work |
| **LafB_pstI_R** | GTTGTTCTGCAGGATAAGCGGAGATGACCTG |  |  |
| **LafB_F (KpnI)** | GGTACCGTGAAGGGGGACGTAAGGTG | 1084 | (20) |
| **LafB_R (XbaI)** | TCTAGACTCCAAACCTAGTCCTTGACCT |  |  |
| **pAT28_F** | TAATGCAGCTGGCACGACAGG | 552 | (20) |
| **pAT28_R** | GCGTGATTGCCAAGCACGTCC |  |  |
| **OriF** | CAATAATCGCATCCGATTGCA |  | (40) |
| **KS05seqR** | CCTATTATACCATATTTTGGAC |  |  |

**Table S3.** Glc_1_DAG species identified in lipid extracts, including their retention time, adduct type, and mass-to-charge ratio (m/z).

| **Lipid name** | **Retention time (min)** | **Adduct type** | **m/z** |
| --- | --- | --- | --- |
| Glc_1_DAG 28:1 | 7.03 | [M+NH_4_]^+^ | 690.51 |
| Glc_1_DAG 28:0 | 7.38 | [M+NH_4_]^+^ | 692.52 |
|  | 7.30 | [M+NH_4_]^+^ | 692.53 |
| Glc_1_DAG 29:0 | 7.44 | [M+NH_4_]^+^ | 706.55 |
| Glc_1_DAG 30:2 | 7.09 | [M+NH_4_]^+^ | 716.53 |
| Glc_1_DAG 30:1 | 7.33 | [M+NH_4_]^+^ | 718.55 |
| Glc_1_DAG 30:0 | 7.51 | [M+NH_4_]^+^ | 720.55 |
|  | 7.57 | [M+NH_4_]^+^ | 720.56 |
| Glc_1_DAG 31:1 | 7.57 | [M+NH_4_]^+^ | 732.55 |
|  | 7.49 | [M+NH_4_]^+^ | 732.56 |
| Glc_1_DAG 31:0 | 7.70 | [M+NH_4_]^+^ | 734.58 |
| Glc_1_DAG 32:2 | 7.38 | [M+NH_4_]^+^ | 744.56 |
| Glc_1_DAG 32:1 | 7.59 | [M+NH_4_]^+^ | 746.58 |
| Glc_1_DAG 32:0 | 7.83 | [M+NH_4_]^+^ | 748.59 |
| Glc_1_DAG 33:2 | 7.55 | [M+NH_4_]^+^ | 758.58 |
| Glc_1_DAG 33:0 | 7.93 | [M+NH_4_]^+^ | 762.61 |
| Glc_1_DAG 34:3 | 7.45 | [M+NH_4_]^+^ | 770.58 |
| Glc_1_DAG 34:2 | 7.42 | [M+NH_4_]^+^ | 772.58 |
|  | 7.61 | [M+NH_4_]^+^ | 772.59 |
| Glc_1_DAG 34:1 | 7.83 | [M+NH_4_]^+^ | 774.61 |
| Glc_1_DAG 34:0 | 8.07 | [M+NH_4_]^+^ | 776.62 |
| Glc_1_DAG 35:2 | 7.79 | [M+NH_4_]^+^ | 786.61 |
| Glc_1_DAG 35:1 | 8.01 | [M+NH_4_]^+^ | 788.62 |
| Glc_1_DAG 36:3 | 7.79 | [M+NH_4_]^+^ | 798.61 |
| Glc_1_DAG 36:2 | 7.84 | [M+NH_4_]^+^ | 800.62 |
| Glc_1_DAG 36:1 | 8.08 | [M+NH_4_]^+^ | 802.64 |
| Glc_1_DAG 37:2 | 8.02 | [M+NH_4_]^+^ | 814.64 |
| Glc_1_DAG 38:2 | 8.08 | [M+NH_4_]^+^ | 828.66 |

**Table S4.** List of annotated Glc_2_DAG species in bacterial lipid extracts, including their retention time, adduct type, and mass-to-charge ratio (m/z).

| **Lipid name** | **Retention time (min)** | **Adduct type** | **m/z** |
| --- | --- | --- | --- |
| Glc_2_DAG 28:1 | 6.73 | [M+NH_4_]^+^ | 852.57 |
| Glc_2_DAG 28:0 | 7.04 | [M+NH_4_]^+^ | 854.57 |
| Glc_2_DAG 30:2 | 6.81 | [M+NH_4_]^+^ | 878.58 |
| Glc_2_DAG 30:0 | 7.34 | [M+NH_4_]^+^ | 882.61 |
| Glc_2_DAG 31:1 | 7.25 | [M+NH_4_]^+^ | 894.61 |
|  | 7.18 | [M+NH_4_]^+^ | 894.62 |
| Glc_2_DAG 31:0 | 7.47 | [M+NH_4_]^+^ | 896.63 |
| Glc_2_DAG 32:3 | 6.99 | [M+NH_4_]^+^ | 904.60 |
|  | 6.93 | [M+NH_4_]^+^ | 904.60 |
| Glc_2_DAG 32:2 | 7.12 | [M+NH_4_]^+^ | 906.61 |
| Glc_2_DAG 32:1 | 7.36 | [M+NH_4_]^+^ | 908.63 |
| Glc_2_DAG 32:0 | 7.58 | [M+NH_4_]^+^ | 910.65 |
| Glc_2_DAG 33:2 | 7.30 | [M+NH_4_]^+^ | 920.63 |
| Glc_2_DAG 33:0 | 7.71 | [M+NH_4_]^+^ | 924.66 |
| Glc_2_DAG 34:3 | 7.20 | [M+NH_4_]^+^ | 932.63 |
| Glc_2_DAG 34:2 | 7.39 | [M+NH_4_]^+^ | 934.65 |
| Glc_2_DAG 34:1 | 7.59 | [M+NH_4_]^+^ | 936.66 |
| Glc_2_DAG 34:0 | 7.82 | [M+NH_4_]^+^ | 938.68 |
| Glc_2_DAG 35:3 | 7.46 | [M+NH_4_]^+^ | 946.65 |
| Glc_2_DAG 35:2 | 7.55 | [M+NH_4_]^+^ | 948.66 |
| Glc_2_DAG 35:1 | 7.76 | [M+NH_4_]^+^ | 950.68 |
| Glc_2_DAG 36:4 | 7.35 | [M+NH_4_]^+^ | 958.65 |
| Glc_2_DAG 36:3 | 7.45 | [M+NH_4_]^+^ | 960.66 |
| Glc_2_DAG 36:2 | 7.60 | [M+NH_4_]^+^ | 962.68 |
| Glc_2_DAG 36:1 | 7.82 | [M+NH_4_]^+^ | 964.69 |
| Glc_2_DAG 37:2 | 7.77 | [M+NH_4_]^+^ | 976.69 |

**Table S5.** List of strains used in this study.

| ***E. faecium* strain** | **Description** | **DAP MIC (mg/mL)** | **Reference** |
| --- | --- | --- | --- |
| *E. faecium* SM1*lafBc577* | Naturally occurring *lafB* mutant possessing a C>T transition at *lafB*nucleotide 577 | 0.125 | (20) |
| *E. faecium* SM1 | Revertant to a *lafB* wild-type form (T577C) evolved from SM1*lafBc577* | 2 | (20) |
| *E. faecium* SM1*Δ lafB* | Derivative of SM1 harboring a deletion of *lafB* | 0.125 | This work |
| *E. faecium* SM1*lafBc577Δ lafB -* pAT28:: *lafB* | Complementation of *lafB* in *cis* (native chromosomal location) | 2 | This work |
| *E. faecium* SM1-1.23* | Derivative of SM1 | 256 | This work |
| *E. faecium* SM1-1.23*ΔlafB* | Derivative of SM1-1.23 harboring a deletion of *lafB* | 0.125 | This work |
| *E. faecium* SM1-1.23 *ΔlafB* -pAT28:: *lafB* | Complementation of *lafB* in *cis* (native chromosomal location) | 32 | This work |
| *E. faecium* HOU503 | DAP susceptible clinical isolate | 2 | (12) |
| *E. faecium* R496 | DAP resistant clinical isolate | 32 | (12) |
| *E. faecium* R497 | DAP resistant clinical isolate | 16 | (12) |
| *Staphylococcus aureus* HG003 | laboratory *S. aureus* strain | - | (61) |
| *S. aureus* HG003 $\Delta$*ugtP*::*kan^R^* | Derivative of above strain with deleted *ugtP*/*ypfP* gene | - | (31) |

*SM1-1.23 is derivative of daptomycin evolution *in vitro E. faecium* SM1 replicate 1, day 23.
